# Demographic and clinical predictors of treatment outcomes in invasive lobular carcinoma breast cancer: insights from Cox regression analysis

**DOI:** 10.1007/s12672-025-03029-6

**Published:** 2025-07-07

**Authors:** Sonia Kaindal, B. Venkataramana

**Affiliations:** Department of Mathematics, School of Advanced Sciences, Vellore Institute of Technology, Vellore, India

**Keywords:** Survival probability, Cox regression, Log rank test, SEER database

## Abstract

Invasive lobular carcinoma (ILC) ranks as the second most prevalent type of breast cancer after invasive ductal carcinoma (IDC). Understanding the demographics, clinical, and treatment outcomes of patients with ILC is essential for developing personalized treatment strategies. The purpose of this study is to evaluate the risk factors, treatment efficacy, demographics, and effects of marital status on treatment approaches for patients with lobular carcinoma. The data retrieved from the SEER program included 2,085 patients with lobular carcinoma. Descriptive statistics describe their clinical and demographical characteristics, while inferential statistics, including the Pearson chi-square test and Cox regression models, assess treatment outcomes based on age and clinicopathological factors. Among the cohort, 7.9% of patients were aged 30–44, 40.1% were aged 45–59, and 52% were aged 60–74. The analysis indicated that patients aged 45–59 predominantly received radiation therapy, while those aged 60–74 primarily underwent chemotherapy. Compared to older individuals, younger patients demonstrated a more favorable response to chemotherapy (HR = 0.653, 95% CI: 0.261–1.633) and radiation therapy (HR = 0.625, 95% CI: 0.249–1.565). Age at diagnosis was an independent factor in breast cancer of lobular carcinoma. The Cox regression models revealed significant disparities in treatment effects across different age groups and clinicopathological characteristics. The chi-square analysis showed no significant associations for most variables, indicating that unmeasured factors influence chemotherapy and radiation therapy. A frailty model better captures risk factors, improving treatment decision-making and patient outcome analysis. This study emphasizes the need to evaluate demographic and clinical factors in treatment planning for lobular carcinoma patients. The findings suggest that personalized treatment strategies should be developed to address the varying responses to treatment among different patient cohorts.

## Introduction

### Background

Every year, approximately 43,000 new cases of invasive lobular breast cancer are diagnosed. Along with its widespread occurrence, a thorough comprehension of the distinctive biology of lobular breast cancer continues to be unattainable. ILC accounts for approximately 5–15% of all invasive breast malignancies, with a significant rise in occurrences over the last twenty years [1]. Research suggests that ILC tends to occur more frequently in the same (ipsilateral) breast compared to invasive ductal carcinoma (IDC) [2]. Compared to IDCs, pleomorphic lobular breast carcinoma has been associated with higher histologic grade and higher AJCC stage [3]. Although less prevalent than IDC, little research has been conducted on its biological features and long-term clinical outcomes, including response to adjuvant therapy [4].

### Literature review

Univariate analysis of various clinical and pathological factors related to local-regional outcomes, overall survival, and recurrence showed that an ILC diagnosis was not identified as a significant risk factor [5]. This study has analyzed TN-ILC and TN-IDC cases (2010–2018) using survival and regression analyses. TN-ILC patients were older, with lower chemotherapy response rates. While survival was similar, Black race and advanced stage predicted worse outcomes, while treatment improved survival [6].

The National Cancer Database (2010–2014) was used for patients aged 18 and older with stage non-MBC and I–III MBC. Log-rank tests, Kaplan–Meier curves, and Cox regression models assessed distant recurrence risks and overall survival, while likelihood ratio analysis measured BCI’s added prognostic value beyond clinicopathologic factors, and logistic regression identified predictors of MBC diagnosis [7–9].

A recent study on ILC has employed advanced pathological techniques to assess E-cadherin loss and single-file cell growth, high-resolution genomic profiling to define its luminal subtype, and evaluations of emerging therapeutic strategies. These methodological advances, building on trends since McCart Reed et al., enhance understanding of ILC’s unique clinical behavior [10].

Another study by researchers employed the Kaplan–Meier and multivariate Cox regression to analyze survival, while logistic regression assessed chemotherapy response. TN-ILC patients were older and had lower pathological response rates. Survival was similar between groups, but Black race and advanced stage predicted worse outcomes, while treatment improved survival [11].

Using SEER data (2000–2019), the study compared ILC and IDC survival. Propensity score matching balanced clinicopathological differences, while multivariate Cox regression identified independent prognostic factors. Based on these factors, a nomogram predicting one-, three-, and five-year overall survival was constructed [12]. Nomograms predicting overall and cancer-specific survival were developed using key prognostic factors and validated via C-index metrics [13]. Recent study nomogram models employ Cox regression to assess breast cancer prognosis using hematologic markers, tumor subtype, and TNM categorization. Higher concordance indices and decision curve analysis demonstrate that these models predictive performance than TNM staging alone [14].

A retrospective analysis of 11,085 early-stage breast cancer patients from the National Cancer Database (2004–2019) with clinically node-negative, T1-T2, non-metastatic tumors. Log-rank tests, Kaplan–Meier curves, and Cox regression models were used to assess histology’s impact on overall survival [15]. This study reviewed the National Cancer Database (2010–2017) for stage I–III breast cancers, focusing on MIDLC, ILC, and IDC. Univariate tests (chi-square and Wilcoxon rank-sum) and logistic regression were used to assess surgical decisions, while multivariable Cox proportional hazards regression evaluated overall survival outcomes [16].

Understanding the effects of chemotherapy and radiation treatment on Infiltrating lobular carcinoma (ILC) requires an advanced approach, as treatment outcomes are not uniform across all patients in different age groups. This study employs a frailty approach by combining sociodemographic and clinical variables to examine the impact of varying patient age groups on treatment response. Frailty survival models incorporate unobserved risk factors and enhance the precision and interpretability of treatment outcome estimates. The study of our research will help develop individualized treatment plans and enhance the quality of care provided to each patient.

The objectives of the study are as follows:

1. To assess the relationship between demographic factors and treatment access or outcomes using chi-square tests.
2. To evaluate the survival impact of chemotherapy and radiation therapy using Kaplan-Meier estimators and Cox proportional hazards models.
3. To investigate disparities in treatment access, response, and outcomes across demographic and clinical characteristics.
4. To examine how patient-specific factors influence treatment choices using frailty and hierarchical models.

**Fig. 1:**
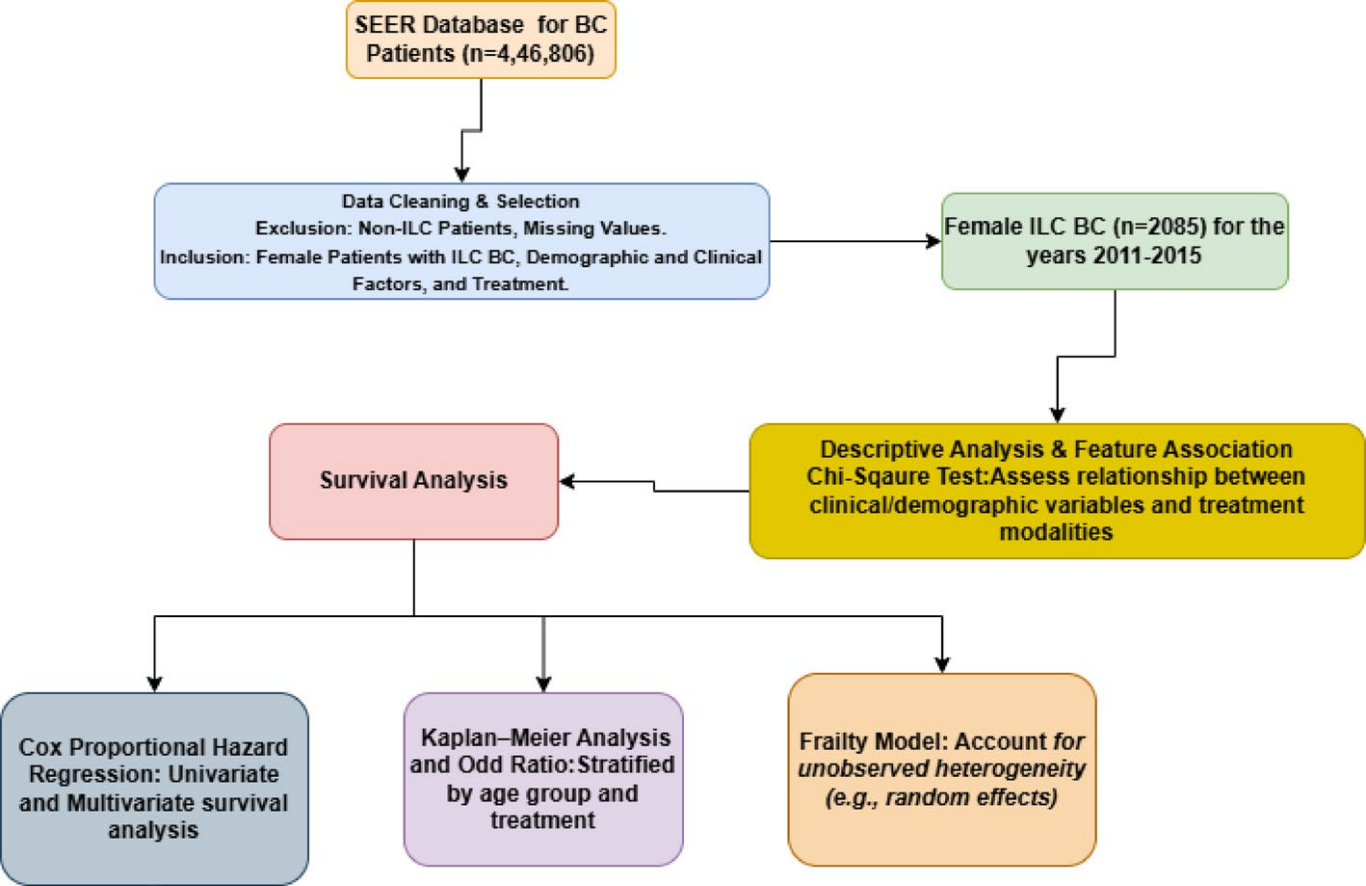
Flow diagram of data analysis and methodology

Figure 1 shows the flowchart of the data analysis and methodology used in this study, outlining each step from data collection to the final analysis.

## Data source

The Surveillance, Epidemiology, and End Results (SEER) Program presents cancer statistics to lower the cancer burden among Americans. The Surveillance Research Program (SRP) of the Division of Cancer Control and Population Sciences at the National Cancer Institute provides funding for SEER.

After cleaning the data from 2011 to 2015, we found 2085 breast cancer patients with lobular carcinoma, survival month, cause of (alive/dead), and vital status(alive/dead): living or deceased, and status of marriage (married, single). Additionally, analysis was conducted based on the characteristics of female breast cancer, which included the year the breast cancer patient diagnosis was made (2011–2015), age at diagnosis (age 30–44, 45–59, 60–74), race (white, black), malignant (Lobular carcinoma), primary site, grade, radiation therapy, and AJCC stage group (7th edition) (IA-IIA, IB-IV, Others). To maintain the integrity and dependability of the study, several rows with a sizable percentage of missing values were eliminated.

We used descriptive statistics to summarize the demographic and clinical characteristic information. Inferential statistics, the Pearson chi-square test assessed, and the t-test compared the demographic and clinical characteristics across different cohorts. The statistical analyses used R, SPSS, MS Excel, and Python.

## Methodology

### Descriptive statistics (Chi-square test)

Chi- square ($\:{\chi\:}^{2}$) test is used to determine if there is a statistically significant association between the age groups (Group I: Age 30–44, Group II: Age 45–59, Group III: Age 60–74) and the categorical sociodemographic factors [17, 18]. The Pearson’s chi-square tests follow [19, 20]:

- Null Hypothesis: There is no association between age groups and demographic and clinical characteristics in ILC breast cancer patients.
- Alternate Hypothesis: There is an association between age groups and demographic and clinical characteristics in ILC breast cancer patients.

The test statistic, chi-square $\:{\chi\:}^{2}$, is determined by applying formula to each of Pearson’s chi-square test

$$\:{\chi\:}^{2}=\sum\:_{i=1}^{n}\left\{\frac{({O}_{i}-{E}_{i}{)}^{2}}{{E}_{i}}\right\}$$

- $\:{O}_{i}$ = Observed frequency (actual no. of patients in the different age groups) in the cell $\:i$.
- $\:{E}_{i}$ = Expected frequency (expected no of patients in the different age groups) in cell *i.*
- $\:i$ = Index representing each observation in the cell.
- n = total no. of observations.

### Product-limit estimator

The patients with ILC were divided into three cohorts to evaluate the survival analysis in different age groups [21]. We examined the overall survival (OS) using a Kaplan-Meier survival plot. The Kaplan-Meier technique estimates the survival function $\:S\left(t\right)$, which shows the likelihood of surviving past a specific time $\:t$:

where the survival time is represented by the random variable $\:T$

$$\:\varvec{S}\left(\varvec{t}\right)=\varvec{P}\left(\varvec{T}>\varvec{t}\right)$$

The Kaplan-Meier estimator $$\\hat :{S}\left(t\right)$$ can be calculated as follows:

$$\\hat :{S}\left(t\right)=\prod\:_{i\le\:t}\left(1-\frac{{d}_{i}}{{n}_{i}}\right)$$

Where:

- The separate event times (such as periods of death or relapse) observed in the dataset are denoted as $\:{\varvec{t}}_{1},{\varvec{t}}_{2},{\varvec{t}}_{3},\dots\:,{\varvec{t}}_{\varvec{m}}$. These are arranged in order such that $\:{\varvec{t}}_{1}<{\varvec{t}}_{2}<\dots\:\dots\:.<{\varvec{t}}_{\varvec{m}}$.
- The quantity of events (such as deaths) that take place at the time $\:{t}_{i}={d}_{i}$.
- The number of patients at risk, that is, the event, is denoted by $\:{n}_{i}$. This is the number of patients shortly before the time $\:{t}_{i}$

The survival probability for each event time $\:{t}_{i}$ by multiplying the prior survival probability by a value that represents the percentage of survivors at that time [22]. The expression $\:1-{d}_{i}/{n}_{i}$ reflects this modification:

- $\:{d}_{i}/{n}_{i}$ represents the percentage of patients who experience the event at $\:t=i$,
- $\:{d}_{i}$ is the likelihood that a person at risk at time $\:{t}_{i}$ survives the past.

### Cox-multivariable regression

A Cox proportional hazard regression model was used to determine factors related to ILC BC patients on the hazard rate of an event. This model generates the hazard ratio (HR) and 95% confidence intervals (CI). Variables with a P-value of 0.05 or less in the univariate analysis were considered possible factors for inclusion in the multivariate analysis. At the time of the most recent follow-up, each patient’s status for breast cancer-specific survival (BCSS) and overall survival (OS) was indicated by a single letter: “yes” if the person had died, or “no” if they were still alive. A two-sided approach was used, and the significant criterion was a P-value of less than 0.05.

- The hazard function $\:h\left(t\right)$ represents the Cox model.
- The risk of dying at the time $\:t$ is known as risk function.

$$\:\mathbf{h}\left(\mathbf{t}\right)={\mathbf{h}}_{0}\left(\mathbf{t}\right){\mathbf{e}}^{\left({\mathbf{b}}_{1}{\mathbf{x}}_{1}+{\mathbf{b}}_{2}{\mathbf{x}}_{2}+\dots\:+{\mathbf{b}}_{\mathbf{p}}{\mathbf{x}}_{\mathbf{p}}\right)}$$

The survival time is denoted by t. The hazard function $\:h\left(t\right)\:$is based on the collection of variables $\:\varvec{p}\left({\varvec{x}}_{1},{\varvec{x}}_{2},\dots\:,{\varvec{x}}_{\varvec{p}}\right)$.

The coefficients $\:\left({\varvec{b}}_{1},{\varvec{b}}_{2},\dots\:,{\varvec{b}}_{\varvec{p}}\right)$ measure the impact of covariates.

The term $\:{h}_{0}$ is called the baseline hazard.

Hazard function for $\:K$ patients

$$\:{\mathbf{h}}_{\mathbf{k}}\left(\mathbf{t}\right)={\mathbf{h}}_{0}\left(\mathbf{t}\right){\mathbf{e}}^{\sum\:_{\mathbf{i}=1}^{\mathbf{n}}\varvec{\upbeta\:}{\mathbf{x}}_{\mathbf{i}}}$$.

Hazard function for $\:{K}^{{\prime\:}}$ patients

$$\:{\mathbf{h}}_{{\mathbf{k}}^{\mathbf{{\prime\:}}}}\left(\mathbf{t}\right)={\mathbf{h}}_{0}\left(\mathbf{t}\right){\mathbf{e}}^{\sum\:_{\mathbf{i}=1}^{\mathbf{n}}\varvec{\upbeta\:}{\mathbf{x}}_{\mathbf{i}}^{\mathbf{{\prime\:}}}}$$.

The hazard ratio for these two patients

$$\:\frac{{\mathbf{h}}_{\mathbf{k}}\left(\mathbf{t}\right)}{{\mathbf{h}}_{{\mathbf{k}}^{\mathbf{{\prime\:}}}}\left(\mathbf{t}\right)}=\frac{{\mathbf{h}}_{0}\left(\mathbf{t}\right){\mathbf{e}}^{\sum\:_{\mathbf{i}=1}^{\mathbf{n}}\varvec{\upbeta\:}{\mathbf{x}}_{\mathbf{i}}}}{{\mathbf{h}}_{0}\left(\mathbf{t}\right){\mathbf{e}}^{\sum\:_{\mathbf{i}=1}^{\mathbf{n}}\varvec{\upbeta\:}{\mathbf{x}}_{\mathbf{i}}^{\mathbf{{\prime\:}}}}}=\frac{{\mathbf{e}}^{\sum\:_{\mathbf{i}=1}^{\mathbf{n}}\varvec{\upbeta\:}{\mathbf{x}}_{\mathbf{i}}}}{{\mathbf{e}}^{\sum\:_{\mathbf{i}=1}^{\mathbf{n}}\varvec{\upbeta\:}{\mathbf{x}}_{\mathbf{i}}^{\mathbf{{\prime\:}}}}}$$

Where:

- $\:{\text{h}}_{\text{k}}\left(\text{t}\right)$ represents the hazard rate for patient$\:\:K$ at time $\:t$.
- $\:{\text{h}}_{0}\left(\text{t}\right)$ represent the baseline hazard function.
- $\:{\mathbf{e}}^{\sum\:_{\mathbf{i}=1}^{\mathbf{n}}\varvec{\upbeta\:}{\mathbf{x}}_{\mathbf{i}}}$ represents the exponentiated linear predictor.
- $\:\varvec{n}$ represents the total number of covariates in the model.

### Frailty model

The frailty model is an extension of the Cox Proportional Hazards model that was developed to take into account unobserved heterogeneity or clustering in survival data that cannot be explained by the covariates that were assessed. Frailty models employ a random effect *Z* to address unmeasured variation among individuals. This modifies the hazard function as follows:

$$\:\:\:\:\:\:\:\:\:\:\:\:\:\:\:\:\:\:\:\:\:\:\:\:\varvec{h}\left(\varvec{t}\right|\varvec{X},\varvec{Z})=\:{\varvec{h}}_{0}\varvec{t}\varvec{exp}\left({\varvec{\beta\:}}_{1}{\varvec{X}}_{1}+{\varvec{\beta\:}}_{2}{\varvec{X}}_{2}+\dots\:\dots\:\dots\:+{\varvec{\beta\:}}_{\varvec{p}}{\varvec{X}}_{\varvec{p}}+\varvec{\alpha\:}\varvec{Z}\right)$$

*Where*:

- $\:\varvec{h}\left(\varvec{t}|\varvec{X},\varvec{Z}\right)$ is the $\:HF$ at time $\:t$ given covariate values $\:X$ and a frailty term $\:Z$.
- $$\:{\varvec{h}}_{0}\varvec{t}$$ is the $$\:BHF$$.
- $\:{\varvec{\beta\:}}_{1},{\varvec{\beta\:}}_{2}\dots\:\dots\:\dots\:..{\varvec{\beta\:}}_{\varvec{p}}$ Are the regression coefficients associated with covariates $\:{\varvec{X}}_{1},{\varvec{X}}_{2},\dots\:\dots\:\dots\:{\varvec{X}}_{3}$
- $\:\varvec{\alpha\:}$ denotes the frailty parameter that signifies the influence of random effects on the hazard.
- $\:\varvec{Z}$ represents the frailty term, which is presumed to adhere to a specific distribution.

## Results and discussion

### Demographic and clinical characteristics of lobular carcinoma breast cancer patients by age group

Our study included a total of 2,085 breast cancer patients diagnosed with lobular carcinoma. Among these, 7.9% were between the age group of 30 and 44, 40.1% were aged 45 to 59, and 52% were in the 60 to 74 age group. The average age of patients ranged from 45 to 69 years. Tables 1 and 2 summarize the frequency and proportion of some clinical and demographic features of breast cancer patients with lobular carcinoma, grouped by age. The relationships between these groups were analyzed using the chi-square test, revealing significant differences in factors of race, AJCC stage group, laterality, marital status, tumor grade, primary site, chemotherapy, and radiation therapy (*p* < 0.05).

Patients with lobular carcinoma in different age groups exhibited varying patterns of treatment. More lobular carcinoma patients tended to receive radiation therapy in the age group (45.5%, 45.5%, and 43.2%, respectively, 30–44, 45–59, and 80 − 74). Women aged 45–59 were more likely to undergo beam radiation therapy, while those aged 60–74 were more likely to receive chemotherapy, with this older group comprising the majority of chemotherapy recipients. There were also cases where patients either refused or were recommended radiation and chemotherapy, but their treatment status remained unknown. Furthermore, a higher proportion of patients with lobular carcinoma in the 60–74 age group were white (87%). Grade II was common in all age groups, and patients 30–44 years were significantly more likely to be single. In contrast, 54. 7% of those 45–59 years of age were married. Furthermore, 37.2% of tumors in the 45–69 age group were located in the upper outer quadrant of the breast.

**Table 1:** Demographic and clinical characteristics of breast cancer

Sociodemographic and Clinical Characteristics	Group I (Age 30–44)	Group II (Age 45–59)	Group III (Age 60–74)	Total
Race Recode				
White	132 (80%)	695 (83%)	909 (83.9%)	1736 (83.3%)
Black	16 (9.7%)	58 (6.9%)	91 (8.4%)	165 (7.5%)
Others	17 (10.3%)	83 (9.9%)	84 (7.7%)	184 (8.8%)
Marital Status				
Married	83 (50.3%)	457 (54.7%)	525 (48.4%)	1065 (51.1%)
Single	26 (15.8%)	112 (13.4%)	139 (12.8%)	277 (13.3%)
Others	56 (33.9%)	267 (31.9%)	420 (38.7%)	743 (35.6%)
AJCC Stage Group				
IA-IIA	74 (44.8%)	420 (50.2%)	514 (47.4%)	1008 (48.3%)
IB-IV	88 (53.3%)	389 (46.5%)	529 (48.8%)	1006 (48.2%)
Others	3 (1.8%)	27 (3.2%)	41 (3.8%)	71 (3.4%)
Grade				
Grade I	49 (29.7%)	257 (30.7%)	328 (30.3%)	634 (30.4%)
Grade II	89 (53.9%)	444 (53.1%)	562 (51.8%)	1095 (52.5%)
Grade III	10 (6.1%)	64 (7.7%)	76 (7%)	150 (7.2%)
Grade IV	0	0	2 (0.2%)	2 (0.1%)
Unknown	17 (10.3%)	71 (8.5%)	116 (10.7%)	204 (9.8%)
Primary Site				
Nipple	0	3 (0.4%)	4 (0.4%)	7 (0.3%)
Central Portion	11 (6.7%)	37 (4.4%)	51 (4.7%)	99 (4.7%)
Upper-Inner Quadrant	26 (15.8%)	82 (9.8%)	110 (10.1%)	218 (10.5%)
Lower-Inner Quadrant	6 (3.6%)	30 (3.6%)	48 (4.4%)	84 (4.0%)
Upper-Outer Quadrant	57 (34.5%)	311 (37.2%)	339 (31.3%)	707 (33.9%)
Lower-Outer Quadrant	9 (5.5%)	61 (7.3%)	75 (6.9%)	145 (7.0%)
Axillary Tail	0	4 (0.5%)	9 (0.8%)	13 (0.6%)
Overlapping Lesion	30 (18.2%)	181 (21.7%)	235 (21.7%)	446 (21.4%)
Breast, NOS	26 (15.8%)	127 (15.2%)	213 (19.6%)	366 (17.6%)

**Table 2:** Treatment characteristics of breast cancer

Treatment Characteristics	Group I (Age 30–44)	Group II (Age 45–59)	Group III (Age 60–74)	Total
Chemotherapy				
No and Unknown	99 (60%)	585 (70%)	773 (71.3%)	1457 (69.9%)
Yes	66 (40%)	251 (30%)	311 (28.7%)	628 (30.1%)
Radiation Therapy	75 (45.5%)	380 (45.5%)	468 (43.2%)	923 (44.3%)
Radiation Beam	5 (3.0%)	10 (1.2%)	17 (1.6%)	32 (1.5%)
Radiation	1 (0.6%)	1 (0.1%)	2 (0.2%)	4 (0.2%)
Refused, Recommended, and Unknown	84 (50.9%)	445 (53.2%)	597 (55%)	1126 (54%)

### Univariate and Multivariate regression analysis of radiation and chemotherapy treatment among breast cancer patients by age group

To compare the clinicopathological characteristics of patients with lobular carcinoma breast cancer of different ages, univariate and multivariate Cox regression models were used to evaluate the impact of factors in three age groups. The follow-up period for this cohort ranged from 1 to 71 months, with a median of 64 months.

The study examined risk factors such as age, race, AJCC stage group, marital status, and grade. The log-rank test was used to determine the relevance of these variables on survival.

Table 3 shows the multivariate Cox regression analysis, We found that age groups and racial categories, AJCC stage group, grade, and marital status were all statistically significantly associated with chemotherapy and radiation therapy (*p* < 0.05). In individuals with lobular carcinoma breast cancer, age at diagnosis was found to be an independent determinant. Compared to older age, younger age had better chemotherapy effect (HR = 0.653, 95% CI: 0.261–1.633) and radiation therapy (HR = 0.625, 95% CI: 0.249–1.565). Patients aged 45 to 59, the hazard ratio for both chemotherapy effect (HR = 1.415, 95% CI: 0.973–2.059) and radiation therapy (HR = 1.386, 95% CI: 0.955–2.013) were elevated compared to other age groups. Although it indicates a reduced treatment effect in this age group.

White patients had hazard ratios of 1.324 (95% CI: 0.611–2.870) for chemotherapy and 1.419 (95% CI: 0.654–3.077) for radiation, while Black patients had hazard ratios of 1.720 (95% CI: 0.660–4.484) for chemotherapy and 1.680 (95% CI: 0.643–4.389) for radiation. For married patients, chemotherapy reduced risk by 65.8% (HR = 0.342, 95% CI: 0.228–0.512), and radiation by 66.5% (HR = 0.335, CI: 0.223–0.503). Single patients saw a 73.3% risk reduction with chemotherapy and 74.7% with radiation (HR = 0.253, CI: 0.121–0.528).

Patients in stages IA-IIA had better chemotherapy survival, 0.592 (CI: 0.182–1.928), suggesting a 40.8% reduction in risk. Radiation therapy in this group had a hazard ratio of 0.731 (95% CI: 0.222–2.401), implying a potential 26.9% risk reduction. For patients in stages IB-IV, chemotherapy had an HR of 0.736 (CI: 0.226–2.394), and radiation therapy had an HR of 0.888 (CI: 0.273–2.882), both had better survival.

Chemotherapy for Grade I tumors has been associated with a significant 61.7% decrease in risk (HR: 0.383, 95% CI: 0.095–1.539). Conversely, radiation therapy in this group shows a slight, non-significant increase in risk (HR: 1.100, CI: 0.543–2.231). In Grade II tumors, chemotherapy demonstrates a significant and noteworthy 80.7% reduction in risk (HR: 0.193, CI: 0.050–0.742), whereas radiation therapy results in a minimal, non-significant risk increase (HR: 1.183, CI: 0.608-2.300). For Grade III tumors, chemotherapy suggests a promising 68.7% risk reduction (HR: 0.313, CI: 0.061–1.601), while radiation therapy indicates a possible, but non-significant, risk increase (HR: 1.327, CI: 0.552–3.190). Tumors located in the central breast region show a pronounced and statistically significant risk increase with both chemotherapy (HR: 2.378, CI: 1.171–4.827) and radiation therapy (HR: 2.382, CI: 1.17–4.821). In contrast, for nipple tumors, both treatments appear to increase risk, but these results remain statistically uncertain. Both therapies show potential, yet non-significant, risk reductions in the upper-inner, lower-inner, and upper-outer breast quadrants. In contrast, the lower outer quadrant suggests a possible increased risk without statistical significance.

**Table 3:** Association of chemotherapy and radiation therapy with breast Cancer characteristics

Sociodemographic and Clinical Characteristics	Chemotherapy HR 95% CI	Radiation Therapy HR (95% CI)
Age		
30–44	0.653 (0.261–1.633)	0.625 (0.249–1.565)
45–59	1.415 (0.973–2.059)	1.386 (0.955–2.013)
Race Recode		
White	1.324 (0.611–2.870)	1.419 (0.654–3.077)
Black	1.720 (0.660–4.484)	1.680 (0.643–4.389)
Marital Status		
Married	0.342 (0.228–0.512)	0.335 (0.223–0.503)
Single	0.267 (0.128–1.633)	0.253 (0.121–0.528)
AJCC Stage Group		
IA-IIA	0.592 (0.182–1.928)	0.731 (0.222–2.401)
IB-IV	0.736 (0.226–2.394)	0.888 (0.273–2.882)
Grade		
Grade I	0.383 (0.95–1.539)	1.100 (0.543–2.231)
Grade II	0.193 (0.050–0.742)	1.183 (0.608-2.30)
Grade III	0.313 (0.061–1.601)	1.327 (0.552–3.190)
Primary Site		
Nipple	1.386 (0.182–10.550)	1.798 (0.237–13.623)
Central Portion	2.378 (1.171–4.827)	2.382 (1.177–4.821)
Upper-Inner Quadrant	0.637 (0.290–1.399)	0.680 (0.310–1.493)
Lower-Inner Quadrant	0.313 (0.073–1.339)	0.328 (0.776–1.410)
Upper-Outer Quadrant	0.565 (0.313–1.018)	0.648 (0.359–1.168)
Lower-Outer Quadrant	1.276 (0.625–2.605)	1.498 (0.734–3.059)
Overlapping Lesions	1.302 (0.749–2.261)	1.344 (0.773–2.335)

**Fig. 2:**
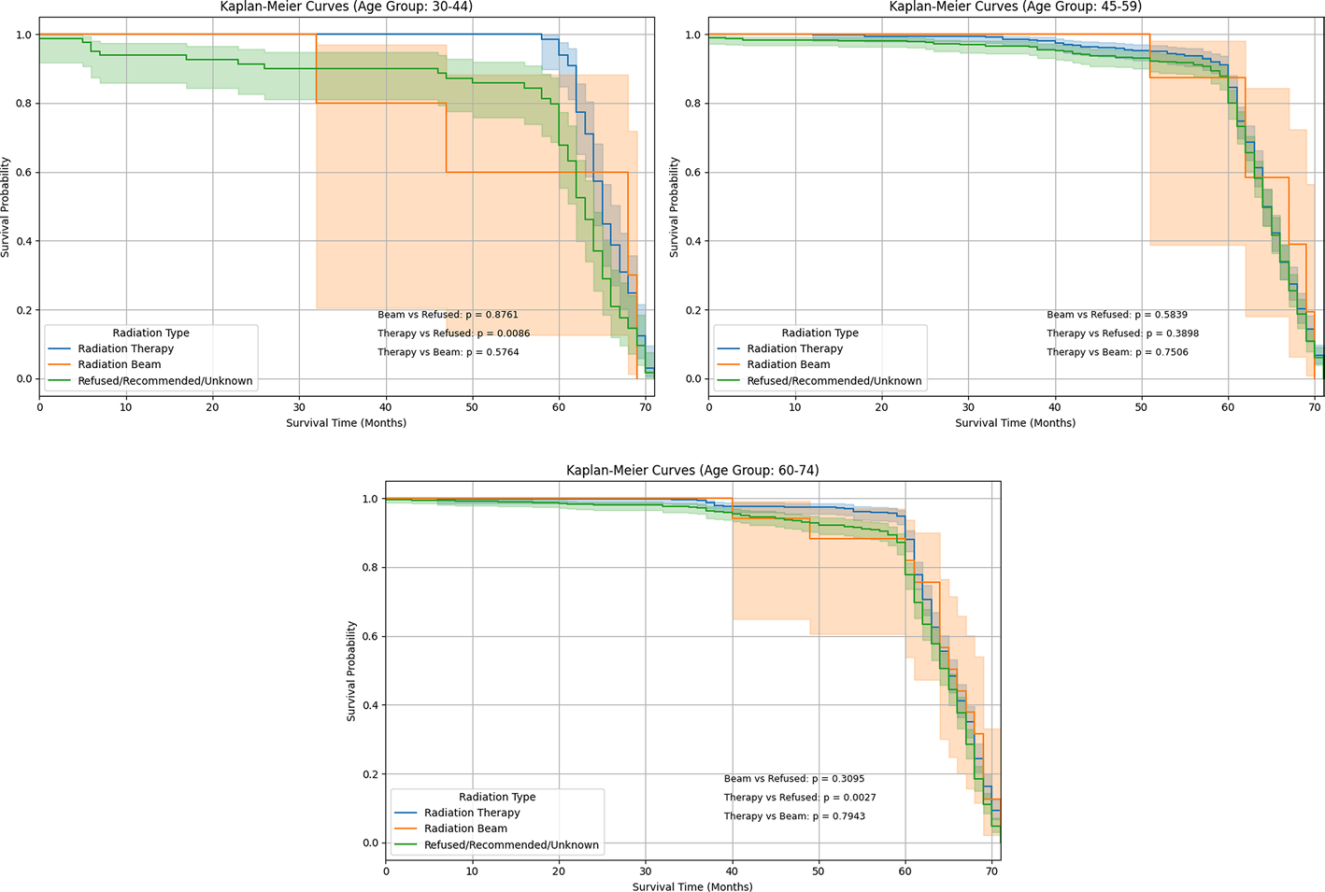
Kaplan-Meier survival for Radiation Therapy patients stratified by age group 30–44, 45–59, and 60–74

Figure 2 shows the Kaplan-Meier survival curve for 30–44 age group patients shows radiation treatment-specific survival probabilities. Radiation therapy (blue line) patients had the highest survival rates during follow-up, indicating better outcomes. Radiation beam therapy (orange line) caused a higher drop in survival around 30–40 months, suggesting less survival. The Refused/Recommended/Unknown (green line) group had less survival than the radiation therapy group but better than the beam therapy group in most time intervals. In the age group of 45 to 59, the Kaplan-Meier survival curve illustrates the effect of radiation therapy on survival for individuals. The standard radiation therapy cohort (blue line) exhibited the highest follow-up survival rates, signifying better results. After 50 months, individuals undergoing radiation beam therapy (orange line) had a decrease in survival and greater confidence intervals, signifying reduced consistency. Radiation therapy enhances long-term survival, however, survival disparities are less pronounced compared to the younger age group.

The Kaplan-Meier curve for the 60–74 age group indicates minimal differences in survival among radiation treatments. Patients undergoing radiation therapy exhibited statistically significantly improved survival rates, particularly after 50 months. The radiation beam cohort exhibited a progressive decrease associated with greater confidence intervals, whereas the Refused/Recommended/Unknown cohort revealed outcomes comparable to the standard therapy group. Radiation therapy provided a slight improvement in survival rates. Our results conclude that a difference in survival between the 45–59 and 60–74 age groups in the radiation therapy was anticipated; the Kaplan-Meier analysis shows a non-significant result, suggesting that the observed variation may be due to chance.

**Fig. 3:**
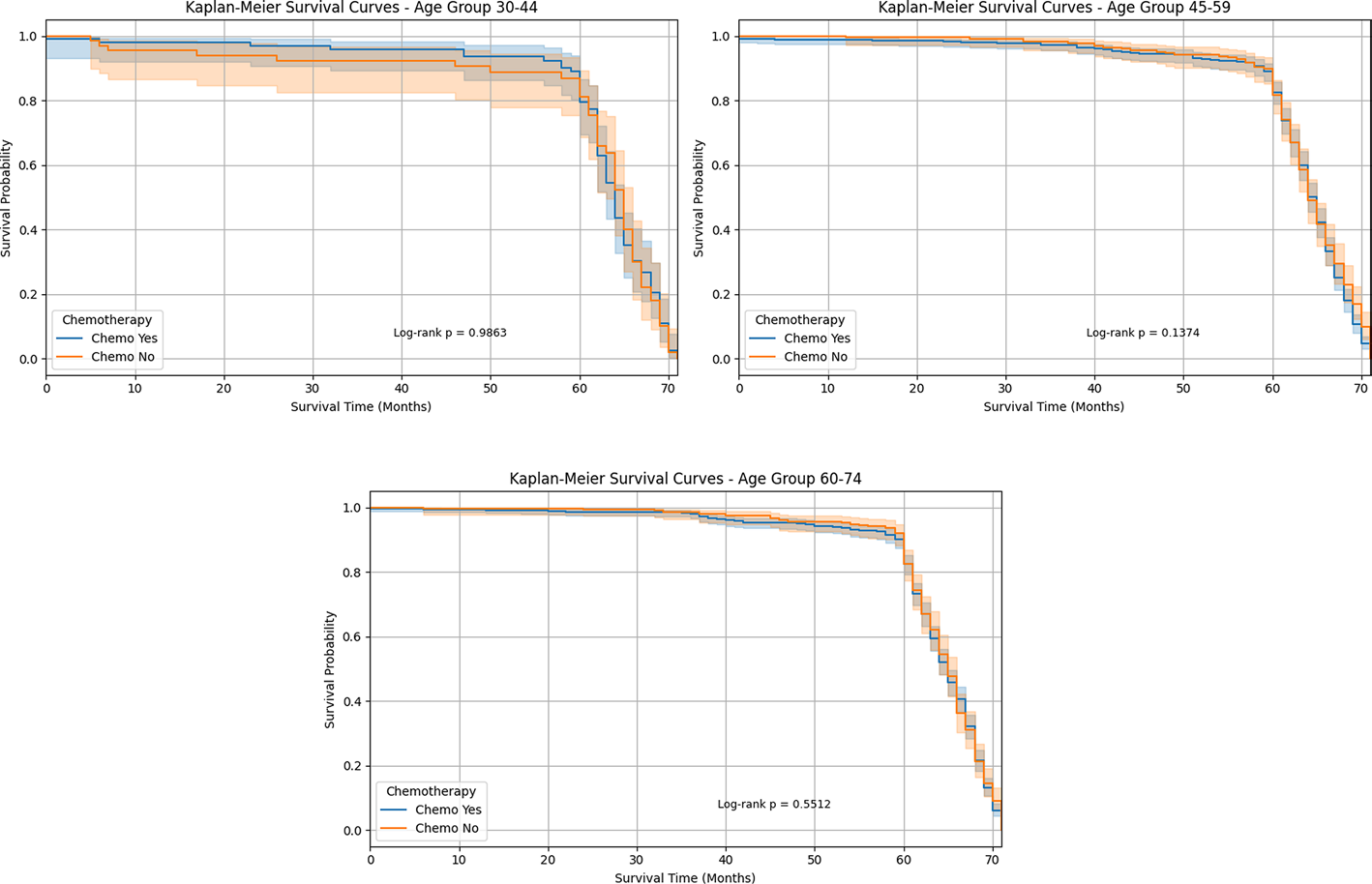
Kaplan-Meier survival for chemotherapy patients stratified by age group 30–44, 45–59, and 60–74

Figure 3 indicates the Kaplan-Meier survival curves for chemotherapy treatment in the age categories 30–44 and 45–59 demonstrate minimal differences in survival outcomes between who receive chemotherapy and those who did not. In the 30–44 age group, individuals who had chemotherapy exhibited an increased survival probability for around 60 months, although the difference is less. In the 45–59 age group, the survival curves for both groups indicate a low effect of chemotherapy on survival within this age group. The age group for 60–74 demonstrates almost equal survival probabilities for patients who underwent chemotherapy compared to those who did not. The two curves almost coincide during the 70-month follow-up period, showing that chemotherapy has a negligible effect on overall survival in this age group. As a result, our finding shows that a difference in survival between all the age groups was anticipated; the Kaplan-Meier analysis shows a non-significant result, suggesting that the observed variation may be due to chance.

Our findings are consistent with prior large-cohort studies, demonstrating a 5-year disease-free survival of 84% and overall survival of 91% for invasive ductal carcinoma [23]. Notably, Black and Hispanic white women with DCIS or LCIS are more prone to advanced-stage disease at diagnosis [24]. We also examined perioperative chemotherapy’s role in ILC prognosis [25], confirming similar invasive cancer risks among Asian, Hispanic, and white women [26]. Additionally, survival outcomes for ILC and IDC were comparable [27]. After adjusting for age, race, and ER status, unmarried women had an 18% higher risk of advanced diagnosis than married women (95% CI: 1.15 to 1.20). These factors increase breast cancer among unmarried women [28]. Acceptance and commitment therapy (ACT) was an efficacious intervention for decreasing depression and enhancing pain acceptance and psychological flexibility in women diagnosed with breast cancer [29]. LCIS incidence increased notably among women aged 50–79 [30]. While some studies report lower survival in ILC post-chemotherapy compared to ER + HER2^−^ IDC [31], conflicting results exist regarding overall ILC survival [32, 33]. Lastly, tumor size and grade remain significant independent predictors of disease-specific survival [34], enhancing our understanding of breast cancer mortality dynamics.

### Impact of frailty effects on demographic and clinical variables over time

The chi-square statistic for both models is 20.64, signifying statistical significance. The integrated log-likelihood model possesses 10 degrees of freedom and a p-value of 0.0238, but the penalized model has 9 degrees of freedom and a p-value of 0.0144, indicating greater significance. The penalized model has a higher AIC (2.64), which means it strikes a balance between model complexity and goodness of fit. The integrated model, on the other hand, has a lower BIC (-53.52).

Table 4 presents the frailty model outcomes. It includes Z-values, P-values, standard errors, coefficient estimates, and hazard ratios. Age (*p* = 0.030) and radiation therapy (*p* = 0.0041) are statistically significant, suggesting they substantially affect the probability of breast cancer occurrences. A negative coefficient for age indicates a potential protective effect, whereas radiation therapy appears to increase the risk.

The frailty model indicates that radiation therapy elevates risk. Conversely, the traditional Cox model in Table 3 indicated that radiation was protective for married women. The difference shows that unobserved factors influence the estimated effect of radiation therapy. To account for potential unobserved heterogeneity and clustering effects related to radiation therapy, we fitted a random effect on the treatment group using a frailty model. The model included fixed effects for age, marital status, radiation, race, cancer stage, laterality, AJCC stage, chemotherapy, and primary tumor site.

**Table 4:** Frailty survival analysis among breast Cancer patients

Sociodemographic and Clinical Characteristics	Coefficient	Exp(Coefficient)	Standard Error	Z-Value	*P*-Value
Age	– 0.0851	0.9184	0.0391	-2.18	0.0300
Marital	– 0.0371	0.9636	0.0272	-1.36	0.1700
Radiation	0.0289	1.0293	0.0101	2.87	0.0041
Race	0.0405	1.0413	0.0401	1.01	0.3100
Stage	0.0093	1.0094	0.0246	0.38	0.7000
Laterality	0.0219	1.0221	0.0486	0.45	0.6500
AJCC Stage	0.0515	1.0528	0.0467	1.10	0.2700
Chemotherapy	– 0.0902	0.9137	0.0554	– 1.63	0.1000
Primary Site	0.0011	1.0011	0.0117	0.09	0.9300

**Table 5:** Chi-Square test results for frailty model

Sociodemographic and Clinical Characteristics	Chi-Square	*P*-Value
Age	0.0104	0.919
Marital	0.2424	0.622
Radiation	3.2654	0.071
Race	2.3730	0.123
Grade	3.4817	0.062
Laterality	0.0123	0.912
AJCC Stage	0.6108	0.435
Chemotherapy	1.4048	0.236
Primary Site	0.2256	0.635
GLOBAL	10.3754	0.321

Table 5 presents the outcomes of the chi-square tests, assessing the overall importance of demographic and clinical variables. None of the variables attain conventional statistical significance (*p* < 0.05). Radiation (*p* = 0.071) and Grade (*p* = 0.062) demonstrate marginal significance. The global test result (*p* = 0.321) indicates that the overall model lacks substantial evidence to reject the null hypothesis, suggesting that the added variables do not significantly enhance its fit collectively.

**Fig. 4:**
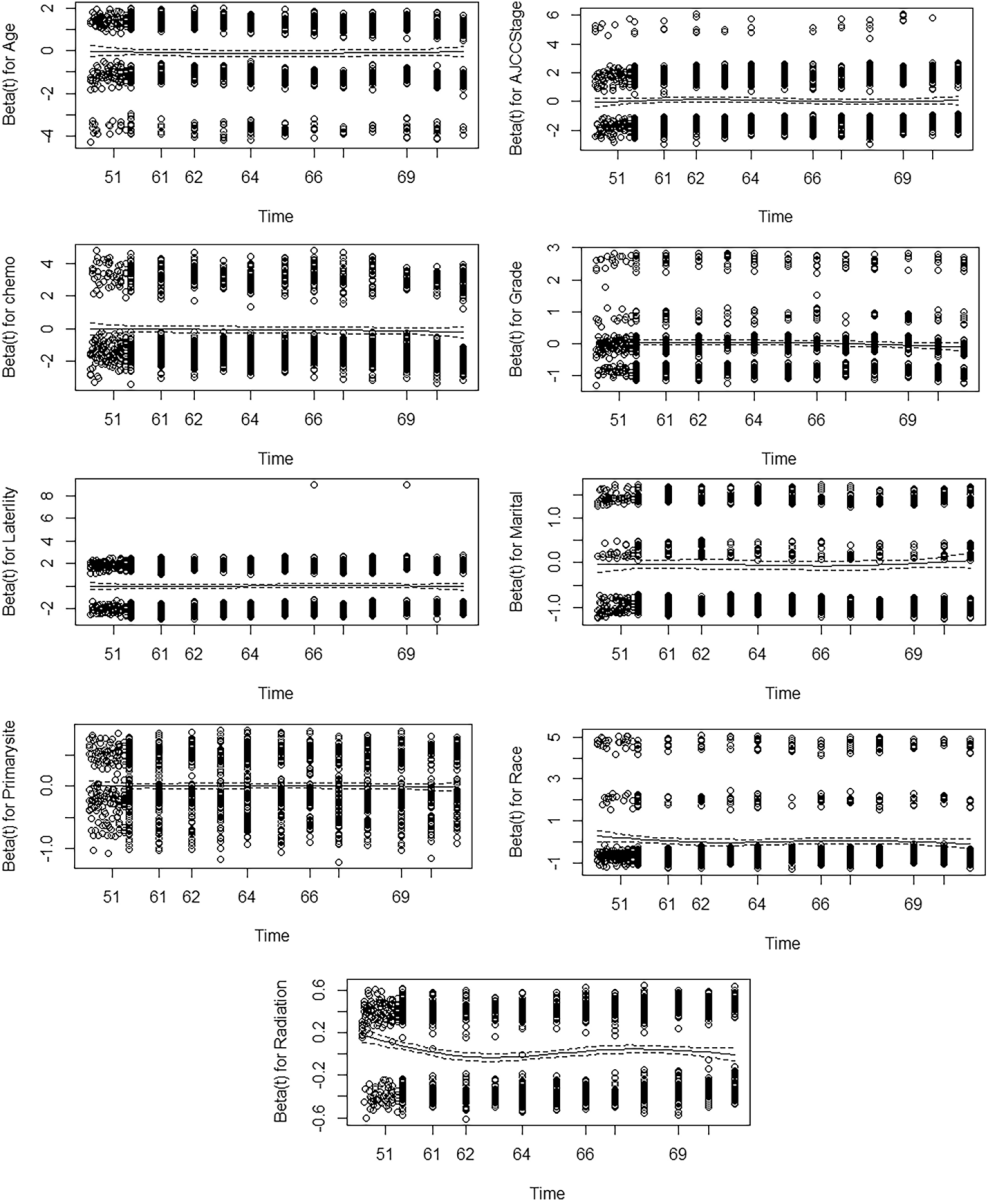
Frailty Effects of Radiation on Survival Over Time

Figure 4 exhibits the random effects (frailty) for age, race, laterality, marital status, radiation, chemotherapy, and the AJCC stage over time within a frailty model. The age effect indicates that the beta estimates for age remain consistently stable and near zero with time. This implies that age exerts neither a significant nor a variable impact on survival beyond 50 months. Similarly, the impact of marriage on survival has nearly diminished over time, suggesting a potentially modest protective effect. The coefficient begins around zero and decreases slightly, signifying a minimal and progressively declining influence of race on survival. The effect of tumor laterality is largely consistent over time and approaches zero. This signifies no significant impact on survival. The impact of chemotherapy is consistently adverse, indicating a survival advantage, though the extent remains comparatively constant across the follow-up period. The effect of grades on the outcome does not change significantly with time. The Radiation Effect shows more variability in how radiation treatment impacts patients. The beta is initially positive but decreases with time, nearing zero. This tendency suggests that the influence of radiation on survival may decrease over time.

The fact that frailty effects are spread out, especially at the beginning, shows that individual risk varies a lot beyond the parameters that were looked at. As time advances, the effects of frailty seem to settle, suggesting that unmeasured heterogeneity may exert a decreasing influence with time. Outliers show that some patients have much higher or lower frailty, highlighting differences in risk that age and AJCC stage alone do not explain.

**Limitations**.

- Our analysis encompassed demographic and clinical parameters; certain significant variables were inaccessible. These encompass comorbidities, genetic predispositions, lifestyle choices (including smoking, alcohol consumption, and dietary habits), and socioeconomic factors, all of which may affect survival and treatment efficacy.
- The Chi-square test showed that many clinical and demographic factors did not have a big effect on the choice of chemotherapy or radiation therapy, which suggests that there were other effects as well. The frailty model emphasizes unobserved heterogeneity yet fails to account for all variability.
- The SEER database lacks information on patient socioeconomic status, which may have influenced treatment plans [35].
- Due to SEER’s lack of specific information regarding endocrine treatment and the distinction between adjuvant and neoadjuvant chemotherapy, our analysis was limited to patients based solely on the available chemotherapy data. We analyzed only individuals who received chemotherapy and who didn’t because of the absence of data on adjuvant chemotherapy or endocrine therapy in the public SEER database. Also, we could only compare individuals who underwent chemotherapy with those who did not, without determining if the latter group may have received alternative treatments such as hormone therapy. This may have resulted in misclassification, particularly for patients with hormone receptor-positive cancer who may have undergone endocrine treatment rather than chemotherapy.
- We did not examine the role of adjuvant chemotherapy or radiotherapy in young women with early-stage breast cancer due to the uncertainty surrounding chemotherapy regimens and the extent of radiotherapy in these patients. Fourth, the data about insurance and socioeconomic status, which have been identified as correlated with young breast cancer-specific survival in prior research, were also absent. Future research needs to take these important factors into account, and we need more prospective randomized controlled studies right away to come up with better methods in this area [36].

**Future Scope**.

- Using frailty modeling in future research can help us better understand the risks that are unique to each patient and make better decisions about how to treat them. More research is required to better understand the biological mechanisms underlying these variations and to create more individualized treatment plans that cater to the unique requirements of various patient groups.
- Future studies ought to focus on long-term follow-ups to evaluate the enduring effects of chemotherapy and other treatments over prolonged durations.
- Examining several frailty distributions (gamma, log-normal, inverse Gaussian) can more accurately represent unobserved variation in breast cancer survival outcomes.
- Future research should investigate the integration of time-dependent clinical variables, such as disease development, therapy alterations, or recurrence, to enhance survival predictions.
- To evaluate the efficacy of emerging treatments like immunotherapy and targeted therapy in comparison to conventional chemotherapy, utilizing Cox proportional hazards and frailty models.

## Conclusion

Our study proposes a survival model that incorporates Cox proportional hazards regression, Kaplan-Meier analysis, and a frailty model, along with interpretability, for patients with invasive lobular carcinoma of the breast. The chi-square analysis indicates that a significant number of clinical and demographic variables do not significantly affect decisions regarding chemotherapy or radiation therapy. This study focuses on how age, race, tumor characteristics, and marital status influence treatment outcomes for individuals with lobular breast cancer. The results of the study shows the importance of including frailty models in survival analysis to account for patient-specific factors that can change how well treatment works. Our research help to provide the personalized therapy regimens that consider these traits may improve therapeutic efficacy and survival rates.

## Data Availability

Data is available on https://seer.cancer.gov/.
